# Therapeutic Effect of Combining Anisodamine With Neostigmine on Local Scar Formation Following Roux-en-Y Choledochojejunostomy in a Novel Rat Model

**DOI:** 10.3389/fphar.2021.700050

**Published:** 2021-09-29

**Authors:** Shao-cheng Lyu, Jing Wang, Wen-li Xu, Han-xuan Wang, Fei Pan, Tao Jiang, Qiang He, Ren Lang

**Affiliations:** Department of Hepatobiliary and Pancreaticosplenic Surgery, Beijing Chaoyang Hospital, Capital Medical University, Beijing, China

**Keywords:** Roux-en-Y choledochojejunostomy, scar formation, anisodamine, neostigmine, inflammatory response

## Abstract

**Background:** The present study aimed to explore the potential effect of combining anisodamine with neostigmine on local scar formation following Roux-en-Y choledochojejunostomy (RCJS) in a novel rat model.

**Methods:** The biliary obstruction model of Sprague Dawley (SD) rats was established in advance, and 54 rats were divided into nine groups randomly (sham operation group, anisodamine group, neostigmine group, combination group, and control group). Anisodamine (25 mg/kg) and neostigmine (50 μg/kg) were injected to the abdominal cavity separately or simultaneously for 1 week since the first day after surgery according to their allocated intervention, while the same amount of saline (0.5 ml) was injected intraperitoneally in the control group. Indexes including body weight, the diameter of the common bile duct, liver function, inflammatory indexes, and the condition of scar formation in different groups at certain time were evaluated in our study.

**Results:** Recovery of liver function (ALT, AST, TB, DB, and GGT) and systematic inflammation indexes (CRP, TNF-α, and IL-1β) in the combination group was prior to that in the control group (*p* < 0.05), while no statistical difference in the serum level of IL-10 was observed among groups. Rats in the combination group represented a wider anastomotic diameter and lower expression of α-SMA and TGF-β1 at anastomotic stoma compared to the control group (*p* < 0.05). Histopathological staining showed slighter proliferation of collagen and smooth muscle fibers in rats’ bile duct wall and less local scar formation at anastomotic stoma compared to the control group.

**Conclusion:** The combination of anisodamine and neostigmine can alleviate local and systemic inflammatory response, promote the recovery of liver function, and reduce scar formation in rats after the RCJS procedure.

## Introduction

While a variety of techniques are available to restore biliary-enteric continuity, the biliary tree is most commonly anastomosed to the jejunum; choledochojejunostomy (CJS) has been widely adopted to biliary surgeries and represents a routine method of biliary reconstruction after surgical resection of pancreatic head carcinoma, cholangiocarcinoma, ampullary carcinoma, duodenal papillary carcinoma, and other tumors (Hirano et al., 2012; Singh and Arora, 2014). Meanwhile, as the morbidity of cancer and radical resection rate increased in recent years, CJS is gaining more and more popularity in clinical settings, among which Roux-en-Y choledochojejunostomy (RCJS) is the most commonly used surgical procedure in clinical practice.

However, intestinal contents and bile reflux secondary to RCJS are inevitable since the valve function of Oddi’s sphincter is no longer available after reconstruction. Continuous stimulation of refluxing fluid can lead to cholangitis and local scar tissue hyperplasia, eventually causing CJS stenosis and even cancer (Kadaba et al., 2017; Bettschart et al., 2002). According to research studies, the incidence rate of CJS stenosis is as high as 13–58% 1 year after CJS (Booij et al., 2018; Yang et al., 2019; Birgin et al., 2020), indicating that inhibiting anastomotic scar formation remains a tough clinical problem. Our previous research studies concluded that the root cause of anastomotic scar formation is the early inflammatory stimulation caused by intestinal content reflux which leads to the activation of fibroblasts and the proliferation of collagen and smooth muscle fibers, ultimately forming a scar (Lyu et al., 2021).

The cholinergic anti-inflammatory pathway has been reported to have modulating effect on inflammatory response in recent years (Ke et al., 2017; Gatta et al., 2020). It transmits anti-inflammatory signals to the reticuloendothelial system (RES) and activates the vagus nerve to release acetylcholine (ACh). The interaction between ACh and α7 nicotinic acetylcholine receptor (α7nAChR) plays a key role in inhibiting inflammatory response and relieving tissue damage. Anisodamine, a widely used belladonna alkaloid that antagonizes mAChR non-selectively, can reinforce the binding force between endogenous ACh and α7nAChR, while neostigmine, a widely used inhibitor against acetylcholinesterase (AChE), can suppress the activity of AChE and prolong the action time of ACh effectively. Their different mechanisms enable them to modulate the cholinergic system in different ways and have synergistic effect when applied simultaneously, increasing inhibition effects on inflammatory reflex effectively (Qian et al., 2015).

Our study aims to figure out the potential effect of combining anisodamine with neostigmine on local scar formation at anastomotic stoma in the novel rat model following RCJS.

## Methods

### Ethics Approval

The study was performed under a project license (No. 2019-D.-304) granted by the Ethics Committee of Beijing Chaoyang Hospital and complied with the institutional guidelines for the care and use of animals.

### Experimental Animals

Specific pathogen free (SPF)-grade male Sprague Dawley (SD) rats aged 6–8 weeks and weighing about 250 g were selected for the present study. The experimental animals were purchased from Beijing Weitong Lihua Animal Experimental (Beijing, China) and were housed at the Medical Research Center of Beijing Chaoyang Hospital (Beijing, China) at normal room temperature with standard chow. The circadian rhythm of the rats was monitored for 12 h.

### Perioperative Management

The rats were fasted (including no access to water) for 6 h before surgery. Surgical anesthesia was performed by an intraperitoneal injection of chloral hydrate (0.5 ml/100 g). After completing the operation, the rats were put into a 37° rewarming table to recover from anesthesia. The rats were not given water for another 12 h after the operation, and normal feeding was resumed after 12 h. All the rats were sacrificed by inferior vena cava bloodletting after the tissue was obtained by laparotomy. All operations were in line with the ethical principles of laboratory animal welfare and approved by the Ethical Member Association of Beijing Chaoyang Hospital affiliated to Capital Medical University.

### Grouping and Intervention

A total of 54 SD rats were randomly divided into nine groups with six rats in each group, which are the sham operation group, anisodamine group (observed for 1 week and 1 month), neostigmine group (observed for 1 week and 1 month), combination group (observed for 1 week and 1 month), and control group (observed for 1 week and 1 month). Rats in the sham operation group received abdominal incision and suture only, while rats in other groups underwent surgery based on the RCJS procedure established in our previous study (Lyu et al., 2021). Anisodamine (25 mg/kg) and neostigmine (50 μg/kg), which had been diluted to 0.5 ml, were injected to the abdominal cavity separately or simultaneously according to their allocated intervention (Li et al., 2014), while the same amount of saline (0.5 ml) was injected to the rats’ abdominal cavity in the control group. The intervention lasted for 1 week since the first day after surgery.

### Measurement and Laboratory Examination

The rats in the sham operation group were sacrificed on the first day after the operation, while other rats were sacrificed at the corresponding time point and reweighed at the time of death, and the diameter of the common bile duct was measured with a vernier caliper. In total, 4 ml blood was collected from the inferior vena cava, and the tissue of RCJS anastomosis was obtained for follow-up examination after the rats were sacrificed. Rats in the sham operation group received same postoperative dispositions as rats in other groups. All blood samples acquired from rats were placed at room temperature for 30 min and then separated by centrifugation (3500 g/min for 10 min; German Sigma Company) to obtain serum. The enzyme-linked immunosorbent assay (ELISA) was applied to measure serum levels of alanine aminotransferase (ALT; Jiancheng, China), aspartate aminotransferase (AST; Jiancheng, China), total bilirubin (TB; Jiancheng, China), direct bilirubin (DB; Jiancheng, China), gamma-glutamyltransferase (GGT; Jiancheng, China), C-reactive protein (CRP; Jianglai Bio, China), tumor necrosis factor-α (TNF-α; Jianglai Bio), interleukin-10 (IL-10; Jianglai Bio, China), and interleukin-1β (IL-1β; Jianglai Bio, China).

### Histological Examination

The tissue of RCJS anastomosis obtained from rats was divided into two pieces averagely. One piece (4-μm section) was fixed in 10% formalin for 48 h, and the tissue specimen was embedded in wax after dehydration, followed by hematoxylin–eosin (HE) staining, Masson’s staining, α-smooth muscle actin (α-SMA) immunohistochemical staining (Abcam, United Kingdom), transforming growth factor-β1 (TGF-β1) immunohistochemical staining (Abcam, United Kingdom), and observation. The mean optical density (MOD) values of Masson’s staining and immunohistochemical staining sections were calculated by Image-Pro Plus 6.0 software; three areas were randomly selected to measure the MOD value of each pathological section under the 200X field of vision, and their average value was taken as the MOD value of the section. The other piece was immediately transferred to liquid nitrogen for preservation and the relative quantitative detection of α-SMA and TGF-β1 by reverse transcription–polymerase chain reaction (RT-PCR). Total RNA was extracted by the TRIzol method (Invitrogen, United States), and cDNA synthesis was carried out by using the SuperScript First Chain Synthesis System (Thermo, United States). The primers were synthesized by raw engineering, and real-time PCR was carried out using SYBR Green PCR Master Mix (Roche, Switzerland).

Primer information was as follows:

GAPDH primer: Forward: 5′-GGCAAGTTCAACGGCACAG-3′

Reverse: 5′-CGCCAGTAGACTCCACGACA-3′

α-SMA primer: Forward: 5′-ATGCTTCTGGACGTACAACTG-3′

Reverse: 5′-GGAATAGCCACGCTCAGTCAG-3′

TGF-β1 primer: Forward: 5′-ATAGCAACAATTCCTGGCGTTACCTT-3′

Reverse: 5′-CCTGTATTCCGTCTCCTTGGTTCAG-3′

### Statistical Analysis

Measurement data are expressed as mean ± standard deviation, following a normal distribution, and median (quartile spacing) in non-normal distribution. For the comparison of measurement data between multiple groups, analysis of variance was used for the normal distribution, while the rank-sum test was used for the non-normal distribution. Comparing the measurement data between the two groups, the *t*-test was used for the normal distribution and the rank-sum test was used for the non-normal distribution. The error diagram was used to describe the observation index. Differences were considered statistically significant when *p* < 0.05. All data were analyzed by SPSS version 22.0 software (IBM, Armonk, NY, United States).

## Results

### Changes of Postoperative Weight and RCJS Anastomotic Diameter in Different Groups

**FIGURE 1:**
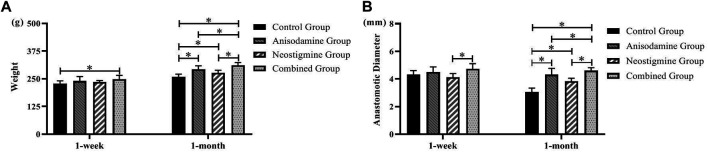
Changes of weight and anastomotic diameter in rats at different time points after the RCJS procedure (* refers to the comparison between the two groups, *p* < 0.05). Comparison of **(A)** weight (g) and **(B)** anastomotic diameter (mm) of rats in different groups at different time points.

The rats’ weight in one-month groups was significantly higher than that in one-week groups (*p* < 0.05), indicating that rats were able to regain their weight gradually after the RCJS procedure. After a week of recovery, only the weight of rats in the combination group exceeded that in the control group (*p* < 0.05). However, after a month of recovery, the weight of rats in all intervention groups including the anisodamine group, neostigmine group, and combination group exceeded that in the control group (*p* < 0.05), and the change was more significant in the combination group. In the control group, the anastomotic diameter of rats in the one-month group was significantly narrower than that in the one-week group, indicating a descent tendency of it as time goes by. Only the diameter of anastomosis in the combination group was wider than that in the neostigmine group after a week of recovery (*p* < 0.05). After a month of recovery, the anastomotic diameter in all intervention groups was greater than that in the control group (*p* < 0.05), and the outcome was much better in the combination group compared to the anisodamine group and neostigmine group (*p* < 0.05) (Figure 1).

### Changes of Liver Function in Different Groups After Surgery

**FIGURE 2:**
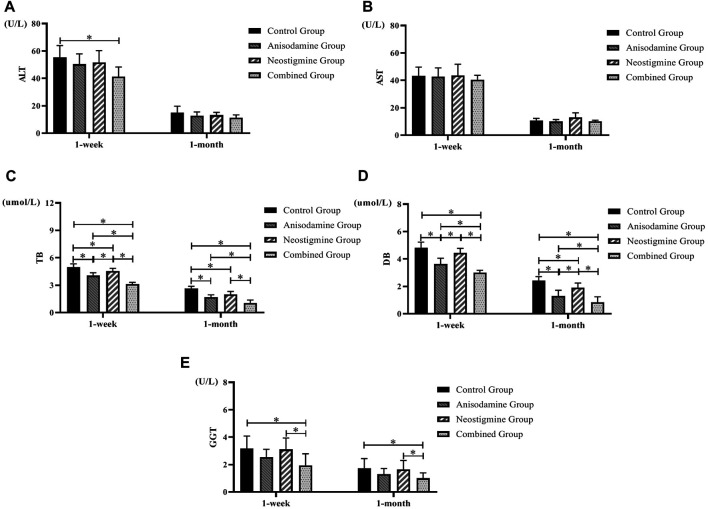
Changes of liver function in rats at different time points after the RCJS procedure (* refers to the comparison between the two groups, *p* < 0.05). Comparison of **(A)** ALT (U/L), **(B)** AST (U/L), **(C)** TB (µmol/L), **(D)** DB (µmol/L), and **(E)** GGT (U/L) at different time points in rats of each group.

Our study indicated that rats were able to gradually regain their liver function over time after the RCJS procedure. In terms of alanine transaminase (ALT), only the rats in the combination group were lower than those in the control group at 1 week after operation (*p* < 0.05); 1 month after operation, there was no significant difference between the experimental groups and the control group. As for aspartate transaminase (AST), there was no significant difference between the experimental groups and the control group at 1 week and 1 month after surgery. At 1 week and 1 month after operation, the level of total bilirubin (TB) and direct bilirubin (DB) in each experimental group was significantly lower than that in the control group (*p* < 0.05); meanwhile, the level in the combination group was lower than that in the anisodamine group and neostigmine group (*p* < 0.05). As for gamma-glutamyl transpeptidase (GGT), only the rats in the combination group were lower than those in the control group at 1 week and 1 month after operation (*p* < 0.05), and the level in the combination group was also lower than that in the neostigmine group (*p* < 0.05). In sum, indexes of liver function recovered more quickly in the combination group than in other groups (Figure 2).

### Changes of Postoperative Inflammatory Indexes in Different Groups

**FIGURE 3:**
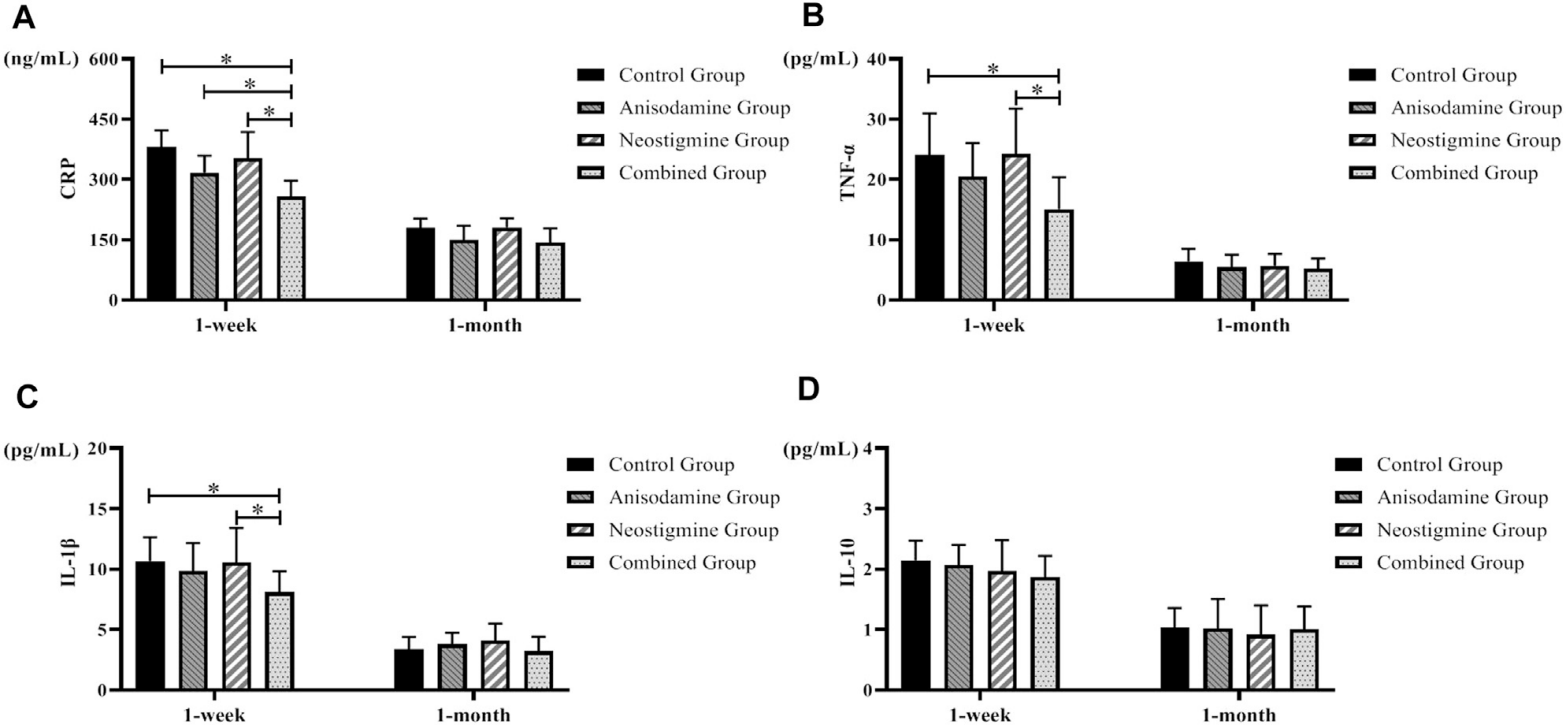
Changes of inflammatory indexes in rats at different time points after the RCJS procedure (* refers to the comparison between the two groups, *p* < 0.05). Comparison of **(A)** CRP (ng/ml), **(B)** TNF-α (pg/ml), **(C)** IL-1β (pg/ml), and **(D)** IL-10 (pg/ml) at different time points in rats of each group.

We observed a gradual recovery in the level of inflammatory indexes in rats after the RCJS procedure. Rats have a lower level of proinflammatory cytokines (CRP, TNF-α, and IL-1β) at 1 week after surgery in the combination group than in the control group and neostigmine group (*p* < 0.05), while no statistical differences were observed in these indexes after 1 month of recovery. As for the level of IL-10, there was no statistical difference between intervention groups and the control group, neither at 1 week after surgery nor at 1 month after surgery. In conclusion, the level of proinflammatory cytokines decreased more rapidly in the combination group, while the level of IL-10 had no statistical difference within different groups at any time points (Figure 3).

### The Pathological Condition of RCJS Anastomotic Stoma After Surgery

**FIGURE 4:**
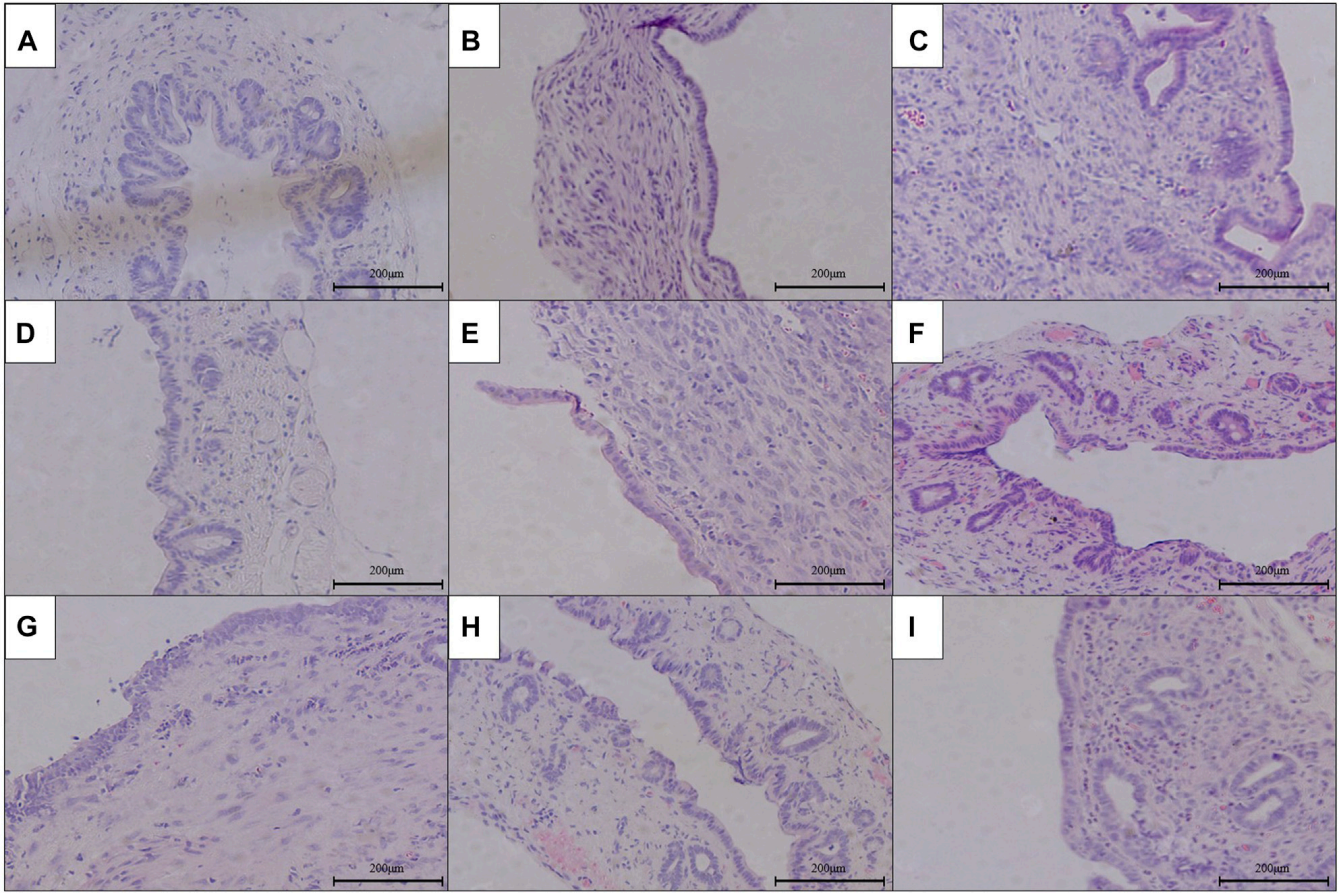
HE staining of anastomotic stoma in different groups after the RCJS procedure (×200). **(A)** Sham operation group. **(B)** Control group at 1 week after the RCJS procedure. **(C)** Control group at 1 month after the RCJS procedure. **(D)** Anisodamine intervention group at 1 week after the RCJS procedure. **(E)** Anisodamine intervention group at 1 month after the RCJS procedure. **(F)** Neostigmine intervention group at 1 week after the RCJS procedure. **(G)** Neostigmine intervention group at 1 month after the RCJS procedure. **(H)** Combination group at 1 week after the RCJS procedure. **(I)** Combination group at 1 month after the RCJS procedure.

**FIGURE 5:**
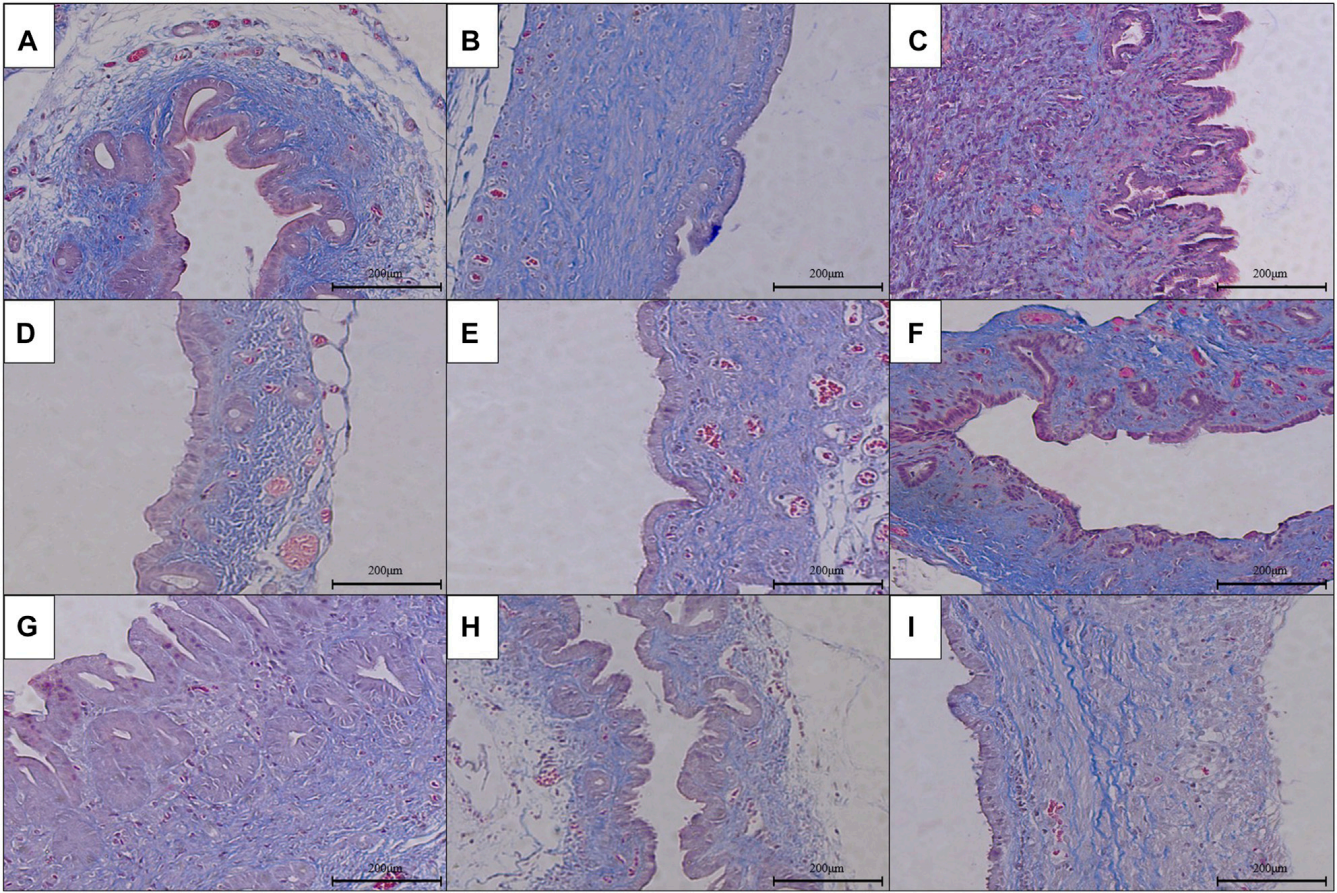
Masson’s staining of anastomotic stoma in different groups after the RCJS procedure (×200). **(A)** Sham operation group. **(B)** Control group at 1 week after the RCJS procedure. **(C)** Control group at 1 month after the RCJS procedure. **(D)** Anisodamine intervention group at 1 week after the RCJS procedure. **(E)** Anisodamine intervention group at 1 month after the RCJS procedure. **(F)** Neostigmine intervention group at 1 week after the RCJS procedure. **(G)** Neostigmine intervention group at 1 month after the RCJS procedure. **(H)** Combination group at 1 week after the RCJS procedure. **(I)** Combination group at 1 month after the RCJS procedure.

**FIGURE 6:**
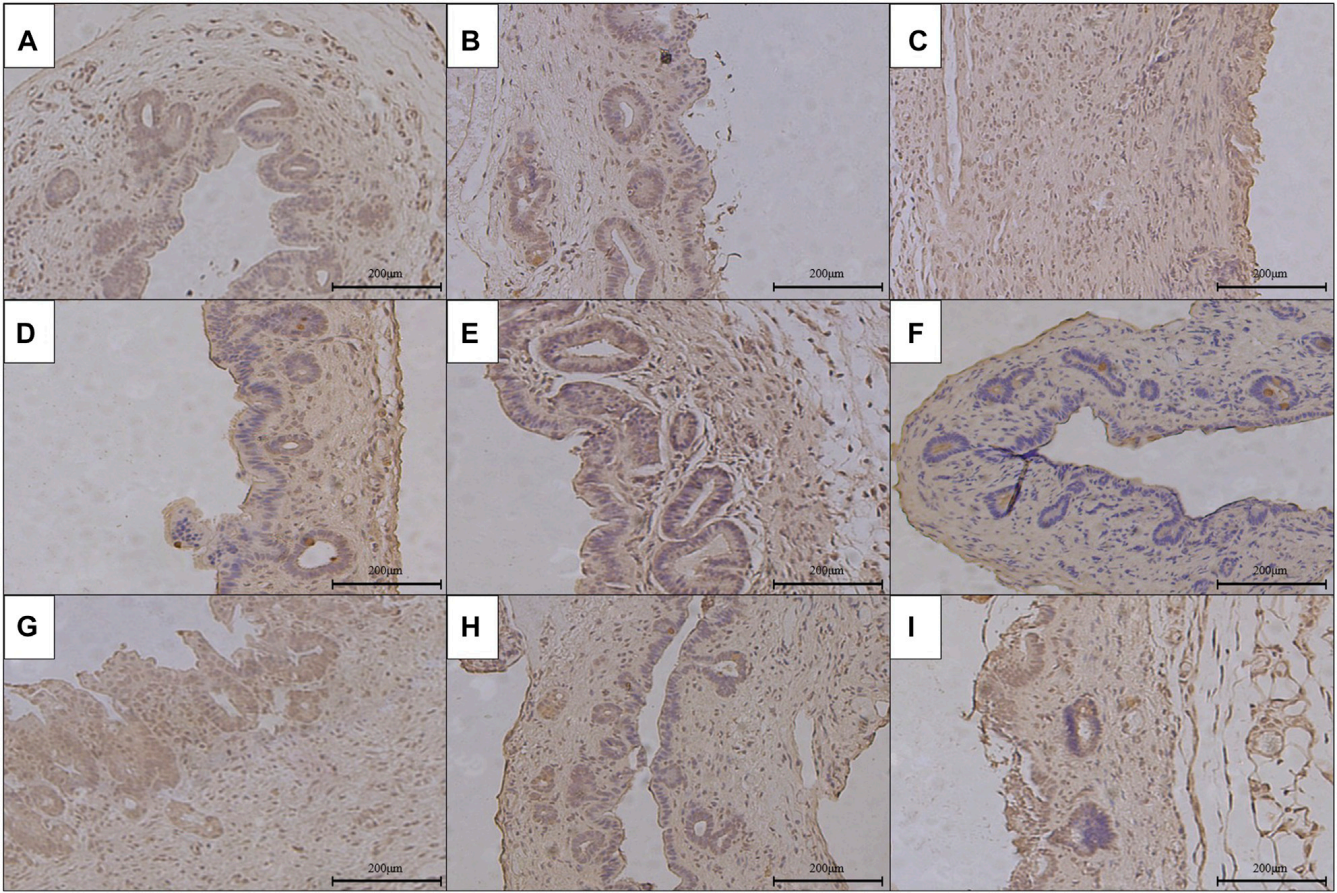
Immunohistochemical staining of α-SMA in the anastomotic stoma after RCJS in different groups (×200). **(A)** IHC staining of α-SMA in the sham operation group. **(B)** IHC staining of α-SMA in the control group at 1 week after the RCJS procedure. **(C)** IHC staining of α-SMA in the control group at 1 month after the RCJS procedure. **(D)** IHC staining of α-SMA in the anisodamine intervention group at 1 week after the RCJS procedure. **(E)** IHC staining of α-SMA in the anisodamine intervention group at 1 month after the RCJS procedure. **(F)** IHC staining of α-SMA in the neostigmine intervention group at 1 week after the RCJS procedure. **(G)** IHC staining of α-SMA in the neostigmine intervention group at 1 month after the RCJS procedure. **(H)** IHC staining of α-SMA in the combination group at 1 week after the RCJS procedure. **(I)** IHC staining of α-SMA in the combination group at 1 month after the RCJS procedure.

**FIGURE 7:**
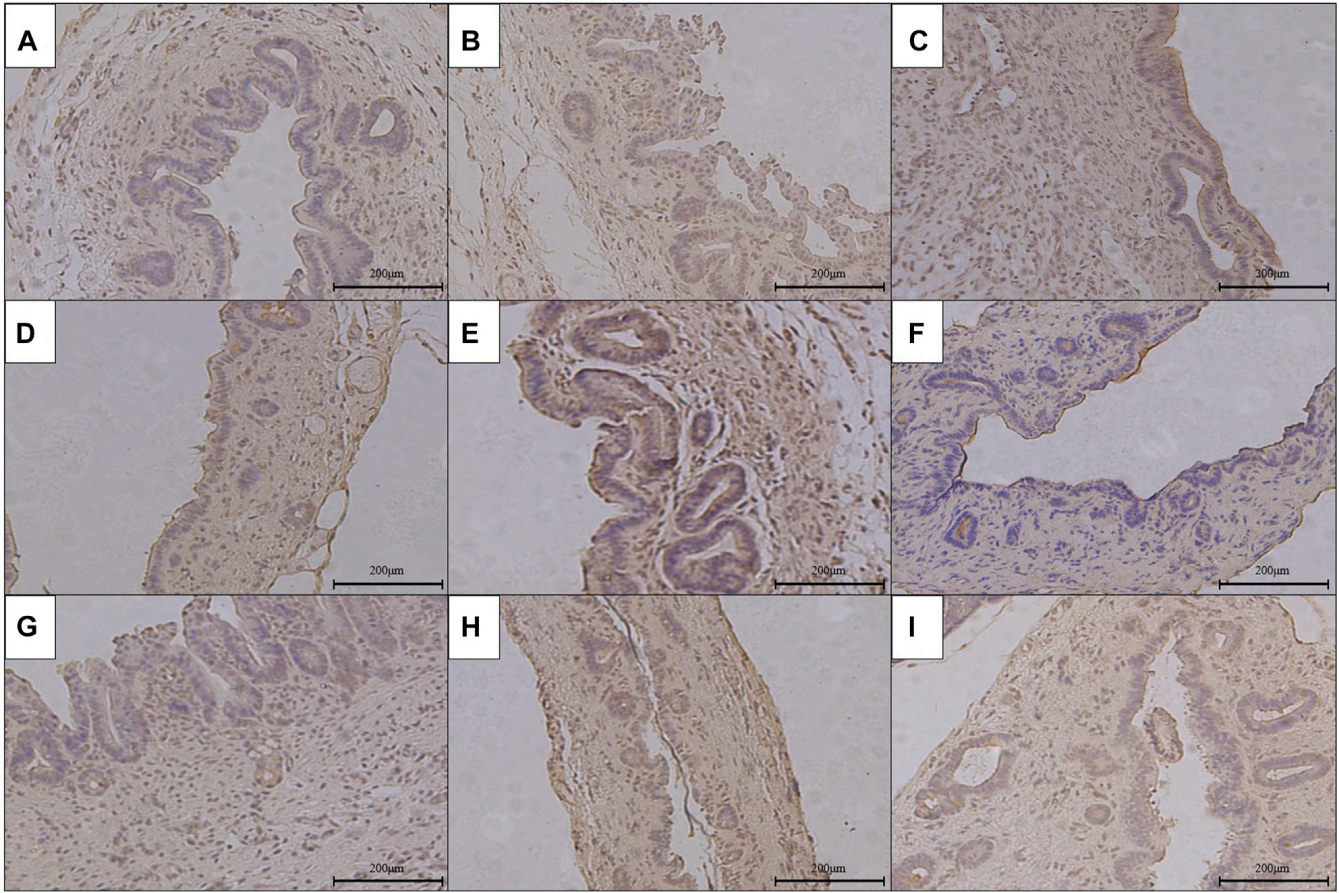
Immunohistochemical staining of TGF-β1 in the anastomotic stoma after RCJS in different groups (×200). **(A)** IHC staining of TGF-β1 in the sham operation group. **(B)** IHC staining of TGF-β1 in the control group at 1 week after the RCJS procedure. **(C)** IHC staining of TGF-β1 in the control group at 1 month after the RCJS procedure. **(D)** IHC staining of TGF-β1 in the anisodamine intervention group at 1 week after the RCJS procedure. **(E)** IHC staining of TGF-β1 in the anisodamine intervention group at 1 month after the RCJS procedure. **(F)** IHC staining of TGF-β1 in the neostigmine intervention group at 1 week after the RCJS procedure. **(G)** IHC staining of TGF-β1 in the neostigmine intervention group at 1 month after the RCJS procedure. **(H)** IHC staining of TGF-β1 in the combination group at 1 week after the RCJS procedure. **(I)** IHC staining of TGF-β1 in the combination group at 1 month after the RCJS procedure.

**FIGURE 8:**
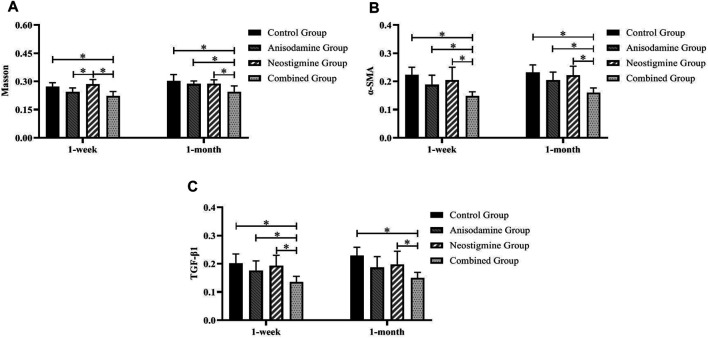
Comparison of the pathological staining MOD of anastomotic stoma in rats at different time points after the RCJS procedure (* refers to the comparison between the two groups, *p* < 0.05). Comparison of MOD values of **(A)** Masson’s staining, **(B)** α-SMA immunohistochemical staining, and **(C)** TGF-β1 immunohistochemical staining at different time points in rats of each group.

The results of HE staining, Masson’s staining, α-SMA immunohistochemical staining, and TGF-β1 immunohistochemical staining at RCJS anastomotic stoma in different groups are shown in Figures 4–8. In the sham operation group, we observed the condition of normal bile duct walls which can be divided into three layers: mucosa layer, muscle layer, and adventitia, which were covered with a monolayer columnar epithelium; normal bile duct walls showed no sign of inflammatory cell infiltration in HE staining. In addition, uniform light blue staining and neatly arranged collagen fibers could be seen in Masson’s staining, while yellow staining of smooth muscle cells along with yellow particles could rarely be found in bile duct walls in α-SMA and TGF-β1 immunohistochemical staining.

However, after the RCJS procedure, inflammatory cell infiltration and incrassation of the bile duct wall were observed at anastomotic stoma and kept deteriorating as time increased. The obvious proliferation and irregular arrangement of collagen and smooth muscle fibers as well as a large amount of yellow-stained smooth muscle cells and yellow particles in the bile duct wall were seen in Masson’s staining and α-SMA and TGF-β1 immunohistochemical staining. Moreover, the proliferation of collagen and smooth muscle fibers along with the expression of yellow particles was at a lower level in the combination group compared to other groups. The results of pathological staining also showed that the MOD values of Masson’s staining and α-SMA and TGF-β1 immunohistochemical staining in the combination group were lower than those in other groups at 1 week and 1 month after operation (*p* < 0.05).

### Relative Quantitative Detection of PCR at RCJS Anastomotic Stoma in Different Groups

**FIGURE 9:**
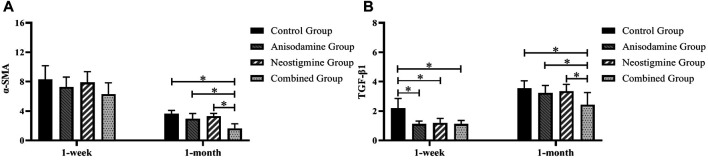
Changes of the expression levels of α-SMA and TGF-β1 in rats at different time points after the RCJS procedure (* refers to the comparison between the two groups, *p* < 0.05). Comparison of **(A)** α-SMA gene expression and **(B)** TGF-β1 gene expression among different groups of rats at different time points.

By comparing the expression level of α-SMA and TGF-β1 at 1 week and 1 month after surgery, we found that, after a week of recovery, the level of TGF-β1 in all intervention groups was lower than that in the control group (*p* < 0.05), while the level of α-SMA showed no statistical difference among the groups. At a month after surgery, only the rats in the combination group had a lower expression level of α-SMA and TGF-β1 compared to those in the other groups (*p* < 0.05) (Figure 9).

## Discussion

The RCJS procedure is widely adopted to biliary reconstruction in clinical practice (Xu et al., 2014). However, since the procedure inevitably brings destruction to the function of Oddi’s sphincter, intestinal content and bile reflux have always been a huge problem in spite of the great progress we have made in medical techniques, and recurring cholangitis caused by intestinal fluid reflux will cause scar hyperplasia, eventually resulting in CJS stenosis and even canceration (Asano et al., 2016; Dimou et al., 2016). Therefore, effective methods to prevent and control local scar formation are still badly needed and have great significance in clinical practice.

Scar formation is a complicated pathophysiological process in which many cells, tissues, and cytokines are involved and can divided into three overlapping phases, inflammatory phase, cell proliferation phase, and tissue remodeling phase. At the initial stage of scar formation (Kerwin et al., 2014; Yen et al., 2018), inflammation plays a key role in this pathophysiological process and may be a potential target where we can modulate this process effectively. In addition, the effects of inflammation on regeneration and repair have also been studied in various mammalian model systems. During postnatal life, the reparative response of most mammalian organisms does not result in tissue regeneration but in the formation of scars with partial loss of organ function. In contrast, the repair response during the early fetal period is regenerative and scar-less (Colwell et al., 2003). The hallmark of fetal repair is the lack of a typical inflammatory response, suggesting that the absence of inflammation is a prerequisite of regenerative and scar-less repair (Redd et al., 2004). In recent years, numerous genetically modified mouse models have advanced our understanding of how the immune response in postnatal life impacts on regeneration and scar formation. Recently, several review articles were published on this topic (Martin and Leibovich, 2005; Mescher and Neff, 2005; Eming et al., 2007). Taken together, gene-modified mouse models highlight the complex and crucial role of inflammation in controlling the quality of repair; however, further studies are necessary to better understand which processes are positive and which are harmful.

Meanwhile, some reports have pointed out that inflammation reaction has run through the whole process of tissue repair and has played the role like a double sword (Eming et al., 2009; Wang et al., 2019). On the one hand, inflammation can induce wound healing and closing through fibrosis and scar formation and prevent wound infection; on the other hand, inflammation-induced scar healing can hamper tissue regeneration and further may reduce the function of tissue or organ. To sum up, inflammatory cell infiltration can inhibit wound repair and regeneration, resulting in excessive scar formation. Therefore, based on the pathophysiology of scar formation and the negative role of early inflammation in scar formation, we believe that controlling early inflammation is the key step to inhibit scar formation.

It is the recurring inflammatory after the RCJS procedure at anastomotic stoma secondary to intestinal fluid reflux that causes local scar formation and leads to aggravation of anastomotic stenosis after surgery as time goes by, showing proliferation of fibroblasts and myofibroblasts in the bile duct wall as their main performance pathologically (Zhang et al., 2011). Siqueira et al. (2017) asserted that, after receiving end-to-end anastomosis, the proportion of collagen and TGF-β1 in the pig’s common bile duct wall in the operation group was significantly higher than that in the sham operation group while showing active proliferation of fibroblasts in the operation group. Our previous research studies (Lyu et al., 2021) also confirmed that the expression of α-SMA and TGF-β1 increased significantly at an early stage after RCJS; subsequently, the fibroblasts proliferated and transformed to myofibroblasts gradually at anastomotic stoma, finally leading to local scar formation. Due to the lack of effective therapeutic targets and drugs to reverse the proliferation of fibroblasts, it will be impossible to modulate and inhibit local scar formation if fibroblasts proliferate extensively or reach the remodeling stage (Block et al., 2015). Through Masson’s staining, we observed a large amount of irregularly arranged collagen and smooth muscle fibers as well as local scar formation in the bile duct wall 1 month after the RCJS procedure in rats. These findings indicated that it may be possible to prevent local scar formation at anastomotic stoma if we can reduce local inflammatory response and inhibit the activation of fibroblasts at an early stage.

Numerous studies (Ren et al., 2017; Shao et al., 2019) have shown that the cholinergic pathway can promote the release of ACh through modulating the efferent vagus nerve. By interacting with α7nAChR distributed on the surface of macrophages, neutrophils, T-cells, and dendritic cells, increasing ACh can act on nucleus and mitochondria through a variety of signal pathways including JAK2-STAT3 and P13K-Akt and subsequently interfere with the expression and synthesis of proinflammatory cytokines including TNF-α, IL-1β, and IL-6, eventually reducing local inflammatory response.

Ulleryd et al. (2019) reported that the serum level of TNF-α, IL-1β, and IL-6 in rats under stress decreased significantly after activating α7nAChR with its agonist. Xu et al. (2016) observed a dramatic increase in the level of TNF-α and IL-6 in muscular tissue of rats with acute crush syndrome and found that activating α7nAChR could not only decrease the level of TNF-α and IL-6 but also prolong the survival period of rats. Further research studies confirmed that these protective effects against inflammatory response were highly dependent on the JAK2-STAT3 pathway.

Anisodamine, a widely used belladonna alkaloid in clinical practice that antagonizes mAChR non-selectively (Eisenkraft and Falk, 2016), is mainly adopted to relieve abdominal pain caused by gastrointestinal spasm and treat septic shock. Neostigmine (Luo et al., 2018), a widely used inhibitor against AChE that can suppress the activity of AChE and prolong the action time of ACh effectively, is clinically applied to antagonize muscle relaxation effect after surgery and treat patients with myasthenia gravis (MG). When applied jointly, anisodamine can reinforce the binding force between endogenous ACh and α7nAChR, while neostigmine can suppress the activity of AChE. Their different mechanisms enable them to modulate the cholinergic system in different ways and have synergistic effect when applied simultaneously, increasing inhibition effects on inflammatory reflex effectively (Zhou et al., 2014). Sun et al. (2012) discovered that, after injecting anisodamine and neostigmine into endotoxic shock mice, they had a lower level of TNF-α and IL-1β and a higher survival rate compared to those in the control group. However, similar results were not observed in α7nAChR-knockout mice with endotoxic shock after same intervention. Li et al. (2014) focused on therapeutic effects of anisodamine combined with neostigmine in rats after partial hepatectomy and found not only a significant decline in serum levels of TNF-α and IL-1β and mRNA levels of TNF-α, IL-1β, and IL-6 in remnant liver but also a significant improvement in the regeneration rate in the combination group compared to the control group. They concluded that the combination of anisodamine and neostigmine was able to reduce inflammatory response and improve remnant liver regeneration after partial hepatectomy through the cholinergic system.

In the present study, we discovered that applying anisodamine and neostigmine jointly at an early stage after the RCJS procedure is able to decrease the serum level of proinflammatory cytokines, relieving the postoperative inflammation and promoting the recovery of liver function in rats. We also found a significant decline in expression levels of α-SMA and TGF-β1 in anastomotic tissue at an early stage after surgery and detected a milder inflammation at anastomotic stoma in the combination group compared to other groups after a month of recovery. We attributed it to the inhibition effect of the cholinergic system on proinflammatory cytokines instead of influencing expression levels of α-SMA and TGF-β1 in anastomotic tissues directly. By inhibiting the synthesis of proinflammatory cytokines, the cholinergic system reduces the inflammatory reaction and infiltration of inflammatory cells at anastomotic stoma, therefore decreasing the secretion of TGF-β1. Without the stimulation of increasing TGF-β1, the activation, proliferation, and differentiation of fibroblasts will be inhibited effectively, ultimately reducing the formation of anastomotic scar tissue. Through the calculation of the MOD value of pathological staining, we also found that Masson’s staining and α-SMA and TGF-β1 immunohistochemical staining of anastomotic tissue in the combination group were better than those in other groups, and the expression of α-SMA and TGF-β1 genes in the combination group was significantly lower than that in the other groups. Meanwhile, at the same time, our study also found that there was no significant change in postoperative ALT and AST among different groups, but the levels of TB and DB in the combination group were significantly lower than those in other groups; we considered that this was due to the fact that the diameter of the anastomotic stoma was relatively wider in the combination group, which was more conducive to bile excretion after biliary obstruction, so the decrease in bilirubin was more significant.

## Conclusion

In conclusion, our study confirmed that the combination of anisodamine and neostigmine can reduce local and systemic inflammatory response, promote the recovery of liver function, and reduce scar formation at anastomotic stoma in rats after the RCJS procedure. Our conclusion brings forward a new idea for preventing and reducing local scar formation after the RCJS procedure, but much more research studies have to be done to verify its clinical value.

## Data Availability

The raw data supporting the conclusions of this article will be made available by the authors, without undue reservation.
